# Chorea as the First Manifestation of Cerebral Infarction

**DOI:** 10.7759/cureus.7384

**Published:** 2020-03-24

**Authors:** Otto Jesus Hernandez Fustes, Renato Puppi Munhoz, Carlos Arteaga Rodriguez, Olga Judith Hernandez Fustes

**Affiliations:** 1 Neurology, Complexo Hospital de Clínicas - Universidade Federal do Paraná (CHC- UFPR), Curitiba, BRA; 2 Neurology, Morton and Gloria Shulman Movement Disorders Centre, Toronto Western Hospital, University of Toronto, Toronto, CAN; 3 Neurology, Universidade Positivo, Curitiba, BRA; 4 Neurophysiology, Clinica Neurológica das Américas, Curitiba, BRA

**Keywords:** hemichorea, ischemic cerebrovascular disease, movement disorder, basal ganglia

## Abstract

Cerebrovascular disease (CVD), which usually manifests as a focal neurological deficit, is presented here as a movement disorder. This unusual manifestation corresponds to 1% of the CVDs and 8% of the striatal lesions. We report a 37-year-old right-handed woman who developed choreic movements as the first manifestation of an acute stroke. The computed tomography (CT) scan revealed a cortical/subcortical hypodense lesion in the right middle cerebral artery territory. This picture slowly improved and remitted completely after six weeks. Basal ganglia infarcts are crucial for the development of hemichorea, however, in spite of its frequency, movement disorders are disproportionally rare. In the majority of cases, the prognosis is good with spontaneous remission after two to four weeks.

## Introduction

Cerebrovascular disease (CVD) is the leading cause of hospitalization for neurological diseases in adults, being responsible for and corresponding to the first and second causes of death in Latin America [1-2].

A typical clinical presentation is rarely questioned, but sometimes, as in the present report, a focal deficit with a loss of function may be replaced by a positive neurological manifestation. At the same time, what happens is the association of this deficit with others as alterations of the level of consciousness, sensory deficit, dysphagia, and visual fields that allow the doctor to locate the injury.

Focal brain injuries, such as CVD, tumors, trauma, multiple sclerosis, and hypoxia, may be the cause of a number of movement disorders although an anatomical structure is rarely restricted. When the CVD represents the cause of a movement disorder, the temporal relationship may be of three forms: acute (after minutes or days), rather than the recovery of the motor deficit; late (after months or years); with the patient and clinically stable [3].

Movement disorders are unusual manifestations in acute CVD, with a 1% to 8% frequency in patients with striatal lesions [3-5]. Among these disorders, we highlight chorea, which is characterized by involuntary, irregular, rapid movements of small amplitude that can involve any body segment with a distal predominance. These involuntary movements at least incorporate themselves into other intentional ones in the attempt to make them less noticeable. An important feature is that the movement disappears during sleep.

Choreic movements are rare in isolation, always occurring in a spectrum ranging from slow, winding movements to the extent that there are more large, violent movements called ballism [6-9].

Among the etiologies, vascular chorea occupies the third most frequent cause, occurring in 9% of cases. The most common cause is Sydenham's chorea in more than 50% of cases, followed by Huntington's chorea in about 20% of cases [10]. However, sudden and lateralized pictures should be considered of vascular origin until proven otherwise.

The anatomical structures responsible for this dysfunction are the base ganglia, particularly the striated, subthalamic nucleus and its pathways.

In our report, we describe the case of a patient who presented vascular chorea as an acute onset of ischemic brain event. Involuntary movements were initially characterized as hemiballism and over the course of two days became a condition of chorea. We find in the literature rare reports of vascular hemichorea as the first manifestation of stroke [4,11-13].

## Case presentation

A woman of age 37 years was admitted to the emergency service with a history of involuntary movements in the left upper limb of three days of evolution, which, 24 hours before admission, was accompanied by motor dysphasia. With a history of health, neglect, use of drugs, illicit drugs, smoking, alcoholism, or a family history of movement disorders.

In the general physical examination, it presented as only alteration arrhythmic cardiac auscultation. At the neurological examination, he was alert, oriented, presenting large, rapid, involuntary motions, both near and distal, random, in the left upper limbs, associated with left central facial palsy and motor dysphasia.

**Figure 1:**
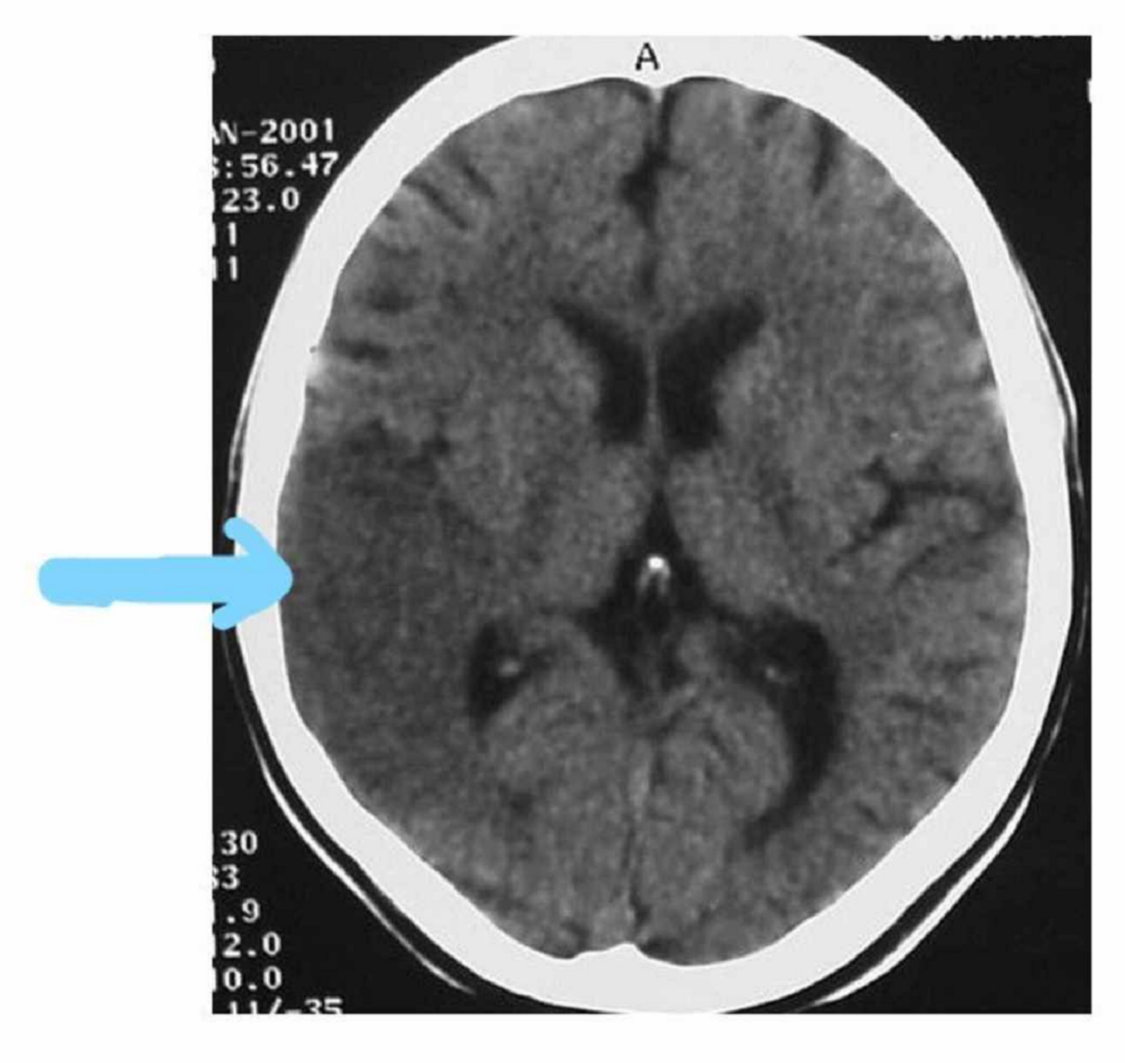
Brain CT with hypodense area compatible with right temporo-parietal ischemia CT: computed tomography

Cranial tomography shows a cortical-subcortical hypodense area compromising the area of ​​the base nuclei in the cerebral hemisphere deriving from the territory of the middle cerebral artery (Figure 1). After atrial fibrillation was verified, the possible etiology of cerebral infarction was of cardioembolic origin. Three weeks after the onset of the condition, there was a complete improvement of speech and reduction of involuntary movements.

## Discussion

We report the case of a patient with a diagnosis of acute vascular disease in the location of the ganglia of the base and cortical-subcortical region of the cerebral hemisphere that developed a disturbance of movement initially characterized as hemiballism and subsequently chorea.

The specific etiologies of vascular lesions described as a cause of chorea are ischemia, hemorrhage, and malformation [14]. The case reported was confirmed by the clinical and complementary investigation of ischemic etiology.

Lesions in the basal ganglia, particularly in the striatum, adjacent white matter in the territory of the middle and posterior cerebral artery, are responsible for movement disorders, as they participate in the regulation of the latter, being part of a circuit that encompasses the motor cortex, striatum, lentiform nucleus, and thalamus. However, although cases of cerebral infarction affecting the base nuclei are frequent, movement disorders secondary to CVD from this location are disproportionately rare [3,5].

In the work of Giroud et al., who studied 20 patients with lenticular infarctions, 16 of them documented putaminal with CT, magnetic resonance imaging (MRI), and single-photon emission computed tomography (SPECT), only five presented some type of movement disturbance, two being chorea, and the remaining three hemidystonia Classically, the lesion responsible for hemiballism or hemichorea is described in the subthalamic nucleus, although it is currently known that the exact topography can be anywhere in the nuclei of the base and cortex-striated pathways [12].

The control of the movements through the ganglia of the base is realized by means of circuits that compose the direct and indirect route. The lesions that affect the integrity of these circuits generate abnormal movements, both of hypokinetic (Parkinsonism) and hyperkinetic (chorea, ballism, and dystonia).

In most patients, the prognosis is good with spontaneous recovery in two to four weeks although some continue to present movements for a long time. In the case reported, the movements remained incapacitating for six weeks of the vascular event. In general, there is a good response to the use of anticholinergics, giving preference to clozapine for having fewer side effects, especially in the elderly who constitute an important part of patients with CVD.

## Conclusions

Our report draws the attention of emergency physicians and neurologists to movement disorders, especially chorea as the initial manifestation of acute ischemic cerebrovascular disease, which requires specific and rapid management to modify the prognosis.
